# Relationship between cell number and clinical outcomes of autologous bone-marrow mononuclear cell implantation in critical limb ischemia

**DOI:** 10.1038/s41598-020-76886-6

**Published:** 2020-11-16

**Authors:** Farina Mohamad Yusoff, Masato Kajikawa, Yuji Takaeko, Shinji Kishimoto, Haruki Hashimoto, Tatsuya Maruhashi, Ayumu Nakashima, S. Fadilah S. Abdul Wahid, Yukihito Higashi

**Affiliations:** 1 Department of Cardiovascular Regeneration and Medicine, Research Institute for Radiation Biology and Medicine (RIRBM), Hiroshima University, 1-2-3 Kasumi, Minami-ku, Hiroshima, 734-8551 Japan; 2 Division of Regeneration and Medicine, Medical Center for Translational and Clinical Research, Hiroshima University Hospital, Hiroshima, Japan; 3 Department of Cardiovascular Medicine, Hiroshima University Graduate School of Biomedical Sciences, Hiroshima, Japan; 4 Department of Stem Cell Biology and Medicine, Hiroshima University Graduate School of Biomedical Sciences, Hiroshima, Japan; 5 Pusat Terapi Sel (Cell Therapy Centre), Universiti Kebangsaan Malaysia (UKM) Medical Centre, Kuala Lumpur, Malaysia

**Keywords:** Peripheral vascular disease, Outcomes research, Regeneration

## Abstract

Cell therapy using intramuscular injections of autologous bone-marrow mononuclear cells (BM-MNCs) improves clinical symptoms and can prevent limb amputation in atherosclerotic peripheral arterial disease (PAD) patients with critical limb ischemia (CLI). The purpose of this study was to evaluate the effects of the number of implanted BM-MNCs on clinical outcomes in atherosclerotic PAD patients with CLI who underwent cell therapy. This study was a retrospective observational study with median follow-up period of 13.5 years (range, 6.8–15.5 years) from BM-MNC implantation procedure. The mean number of implanted cells was 1.2 ± 0.7 × 10^9^ per limb. There was no significant difference in number of BM-MNCs implanted between the no major amputation group and major amputation group (1.1 ± 0.7 × 10^9^ vs. 1.5 ± 0.8 × 10^9^ per limb, P = 0.138). There was also no significant difference in number of BM-MNCs implanted between the no death group and death group (1.5 ± 0.9 × 10^9^ vs. 1.8 ± 0.8 × 10^9^ per patient, P = 0.404). Differences in the number of BM-MNCs (mean number, 1.2 ± 0.7 × 10^9^ per limb) for cell therapy did not alter the major amputation-free survival rate or mortality rate in atherosclerotic PAD patients with CLI. A large number of BM-MNCs will not improve limb salvage outcome or mortality.

## Introduction

Peripheral artery disease (PAD) is a worldwide disease and the number of patients with PAD has increased by nearly a quarter in the past decade. PAD is associated with loss of mobility, functional decline and cardiovascular events, and these changes represent a major public health challenge^1–3^. Critical limb ischemia (CLI) is the end spectrum of PAD. Multiple criteria such as Fontaine classification, Rutherford classification and risk stratification based on wounds, ischemia, and foot infection have been used to determine the severity of CLI for strategic therapies^4–6^. In 20% to 40% of atherosclerotic PAD patients with CLI, there are no suitable interventions or there have been failed previous revascularization therapies, so-called no-option CLI patients, and these patients are at high risk for limb amputation^7^.

The role of cell therapy in patients with CLI has been investigated in a number of subjects^8–13^. Systematic reviews and meta-analyses of randomized, non-randomized and non-controlled studies have shown the efficacy of autologous cell therapy not only with bone-marrow mononuclear cells (BM-MNCs) but also with autologous cells derived from different sources for improving clinical symptoms in patients with CLI^14–17^. It seems that the results of treatment differ depending on the source, severity and regimen. In patients with CLI who were ineligible for surgical or percutaneous revascularization, Rigato et al. showed that autologous cell therapy may have the potential to reduce the risk of major amputation by 36% in primary analysis and improve the probability of wound healing by 59%. Cell therapy was accumulatively found to significantly improve the chances of amputation-free survival by 18% in primary analysis. Reduction in amputation rate and improvement in wound healing rate suggest that cell therapy may be able to modify the natural history of intractable CLI. Reduction in amputation rates was not associated with prolonged survival since the causes of death in patients with severe PAD or CLI are mostly unrelated to PAD^16^. Nevertheless, despite the need for more high-quality placebo-controlled trials, it has been shown that autologous cell therapy has the potential to modify the natural history of intractable CLI.

There is still limited information on the role of implanted cell number in clinical outcomes in atherosclerotic PAD patients with CLI who have undergone cell therapy. Therefore, this study was conducted to evaluate the outcomes in atherosclerotic PAD patients with CLI after undergoing autologous BM-MNC implantation with various numbers of implanted cells to improve amputation-free survival and overall survival.

## Results

### Clinical characteristics

Baseline clinical characteristics of patients who underwent BM-MNC implantation with major amputation and without major amputation are summarized in Table 1. There were no significant differences in the parameters between the two groups. Baseline clinical characteristics of patients who died and those who survived after undergoing BM-MNC implantation are summarized in Table 2. There were significant differences in age and history of myocardial infarction between the two groups. There were no significant differences in other parameters between the two groups. Baseline clinical characteristics of patients who had major adverse cardiovascular events (MACE) and those without MACE after undergoing BM-MNC implantation are summarized in Table 3. MACE is defined as a composite of nonfatal myocardial infarction, nonfatal stroke, and cardiovascular death. There were no significant differences in the parameters between the two groups. The mean number of cells implanted in all of the patients was 1.2 ± 0.7 × 10^9^ (range, 1.0 ± 0.4 × 10^7^- 2.5 ± 0.6 × 10^9^) per limb. The median follow-up period from BM-MNC implantation was 13.5 years (range, 6.8–15.5 years).

**Table 1:** Clinical characteristics of patients with and without major amputation.

Variables	No major amputation (n = 17)	Major amputation (n = 13)	*p* Value
Age, years	69 ± 8	65 ± 10	0.258
Gender, men/women	11/6	8/5	0.858
Body mass index, kg/m^2^	21.2 ± 2.6	23.1 ± 4.3	0.141
**Fontaine category, n (%)**			
III	7 (41)	2 (15)	
IV	10 (59)	11 (85)	
**Rutherford category, n (%)**			
3	1 (6)	0 (0)	
4	7 (41)	2 (15)	
5	6 (35)	9 (69)	
6	3 (18)	2 (15)	
**Medical history, n (%)**			
Hypertension	16 (94)	10 (78)	0.167
Dyslipidemia	10 (59)	8 (61)	0.880
Diabetes mellitus	15 (88)	10 (78)	0.412
Previous myocardial infarction	9 (53)	6 (46)	0.712
Previous stroke	5 (29)	3 (23)	0.696
Chronic kidney disease	8 (47)	6 (46)	0.961
Smoker (pre)	9 (53)	4 (31)	0.221
**Medications, n (%)**			
Anti-coagulant	5 (30)	6 (46)	0.346
Anti-platelets	13 (76)	11 (84)	0.577
Renin angiotensin system inhibitors	10 (59)	6 (46)	0.713
Calcium-channel blockers	9 (53)	4 (31)	0.284
Statins	4 (23)	3 (23)	0.660
Sulfonylurea/metformin/other	8 (47)	3 (23)	0.242
Insulin	4 (23)	2 (15)	0.577

**Table 2:** Clinical characteristics of patients who died and those who survived.

Variables	No death (n = 13)	Death (n = 17)	*p* Value
Age, years	62.7 ± 10.13	70.8 ± 6.7	< 0.05
Gender, men/women	9/4	10/7	0.708
Body mass index, kg/m^2^	21.9 ± 3.3	22.0 ± 3.8	0.973
**Fontaine category, n (%)**			
III	3 (23)	6 (35)	
IV	10 (77)	11 (65)	
**Rutherford category, n (%)**			
3	1 (8)	0 (0)	
4	3 (23)	6 (35)	
5	7 (54)	8 (47)	
6	2 (15)	3 (18)	
**Medical history, n (%)**			
Hypertension	11 (85)	15 (88)	> 0.99
Dyslipidemia	9 (69)	9 (53)	0.465
Diabetes mellitus	11 (85)	14 (82)	> 0.99
Previous myocardial infarction	3 (23)	12 (71)	< 0.05
Previous stroke	5 (38)	3 (18)	0.242
Chronic kidney disease	6 (46)	8 (47)	> 0.99
Smoker (pre)	6 (46)	7 (41)	> 0.99
**Medications, n (%)**			
Anti-coagulant	6 (46)	5 (29)	0.454
Anti-platelets	10 (77)	14 (82)	> 0.99
Renin-angiotensin system inhibitors	9 (69)	7 (41)	0.159
Calcium-channel blockers	7 (54)	6 (35)	0.460
Statins	3 (23)	4 (24)	> 0.99
Sulfonylurea/metformin/other	6 (46)	5 (29)	0.454
Insulin	2 (15)	4 (24)	0.672

**Table 3:** Clinical characteristics of patients with and without 3 points MACE.

Variables	No MACE (n = 21)	MACE (n = 9)	*p* Value
Age, years	65.7 ± 9.8	70.9 ± 6.5	0.103
Gender, men/women	13/8	6/3	> 0.99
Body mass index, kg/m^2^	21.8 ± 2.9	22.2 ± 4.9	0.826
**Fontaine category, n (%)**			
III	7 (33)	2 (22)	
IV	14 (67)	7 (78)	
**Rutherford category, n (%)**			
3	1 (5)	0 (0)	
4	7 (33)	2 (22)	
5	9 (43)	6 (67)	
6	4 (19)	1 (11)	
**Medical history, n (%)**			
Hypertension	18 (86)	8 (89)	> 0.99
Dyslipidemia	13 (62)	5 (56)	> 0.99
Diabetes mellitus	17 (81)	8 (89)	> 0.99
Previous myocardial infarction	8 (38)	7 (78)	0.109
Previous stroke	5 (24)	3 (33)	0.667
Chronic kidney disease	8 (38)	6 (67)	0.236
Smoker (pre)	10 (48)	3 (33)	0.690
**Medications, n (%)**			
Anti-coagulant	7 (33)	4 (44)	0.687
Anti-platelets	17 (81)	7 (78)	> 0.99
Renin angiotensin system inhibitors	11 (52)	5 (56)	> 0.99
Calcium-channel blockers	10 (48)	3 (33)	0.690
Statins	5 (24)	2 (22)	> 0.99
Sulfonylurea/metformin/other	9 (43)	2 (22)	0.419
Insulin	3 (14)	3 (33)	0.329

MACE indicates major adverse cardiovascular events.

Baseline clinical characteristics of patients who underwent BM-MNC implantation with a small number of implanted cells and a large number of implanted cells are summarized in Table 4. The median cell number that was used to differentiate between a low number and a large number was 1.8 ± 0.8 × 10^9^ (range, 1.0 ± 0.8 × 10^7^ – 3.5 ± 0.8 × 10^9^). Age was significantly more advanced in the small cell number group than in the large cell number group. There were no significant differences in other parameters between the two groups. There were no significant differences in overall outcomes in the small cell number and large cell number groups of BM-MNC implantation in this study population (Table 5).

**Table 4:** Clinical characteristics of patients implanted with a small number and a large number of BM-MNCs.

Variables	Small cell number* (n = 15)	Large cell number* (n = 15)	*p* Value
Age, years	71.2 ± 6.9	63.3 ± 9.6	0.016
Gender, men/women	8/7	11/4	0.450
Body mass index, kg/m^2^	21.8 ± 3.6	22.1 ± 3.5	0.837
**Fontaine category, n (%)**			
III	2 (13)	7 (47)	
IV	13 (87)	8 (53)	
**Rutherford category, n (%)**			
3	1 (7)	0 (0)	
4	2 (13)	7 (47)	
5	8 (53)	7 (47)	
6	4 (27)	1 (7)	
**Medical history, n (%)**			
Hypertension	14 (93)	12 (80)	0.598
Dyslipidemia	7 (47)	11 (73)	0.263
Diabetes mellitus	11 (73)	14 (93)	0.329
Previous myocardial infarction	9 (60)	6 (40)	0.466
Previous stroke	5 (33)	3 (20)	0.682
Chronic kidney disease	6 (40)	8 (53)	0.715
Smoker (pre)	5 (33)	8 (53)	0.462
**Medications, n (%)**			
Anti-coagulant	3 (20)	8 (53)	0.128
Anti-platelets	11 (73)	13 (87)	0.651
Renin angiotensin system inhibitors	10 (67)	6 (40)	0.710
Calcium-channel blockers	7 (47)	6 (40)	> 0.99
Statins	3 (20)	4 (27)	> 0.99
Sulfonylurea/metformin/other	5 (33)	6 (40)	> 0.99
Insulin	4 (27)	2 (13)	0.651

*Median cell number between the small number and large number was 1.8 ± 0.8 × 10^9^.

**Table 5:** Overall outcomes of subjects implanted with a small number and a large number of BM-MNCs.

All subjects	Small cell number* (n = 15)	Large cell number* (n = 15)	*p* Value
No deaths, n	7	6	0.290
#Myocardial infarction, n	2	1	0.451
#Heart failure, n	1	2	0.596
#Stroke, n	1	3	0.596
Sepsis, n	1	3	0.302
Malignancy, n	0	1	> 0.99
Upper gastrointestinal bleeding, n	1	0	> 0.99
Lower gastrointestinal bleeding, n	1	0	> 0.99
Intestinal obstruction, n	1	0	> 0.99

^#^ Major cardiovascular events.

*Median cell number between the small number and large number was 1.8 ± 0.8 × 10^9^.

### Implanted cell numbers in the no major amputation and major amputation groups

**Figure 1:**
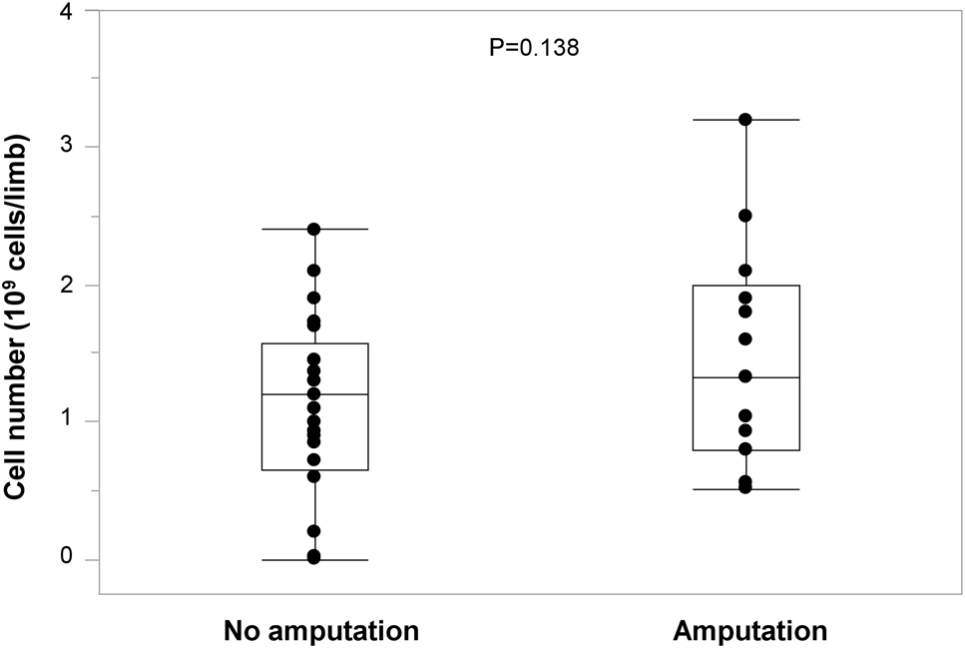
Box plots show implanted cell numbers in the no amputation and amputation groups.

Box plots show implanted cell numbers in the no amputation and amputation groups (Fig. 1). There was no significant difference in the numbers of cells implanted between the no amputation and amputation groups (1.1 ± 0.7 × 10^9^ vs.1.5 ± 0.8 × 10^9^ cells per limb, P = 0.138).

### Implanted cell numbers in the death and no death groups

**Figure 2:**
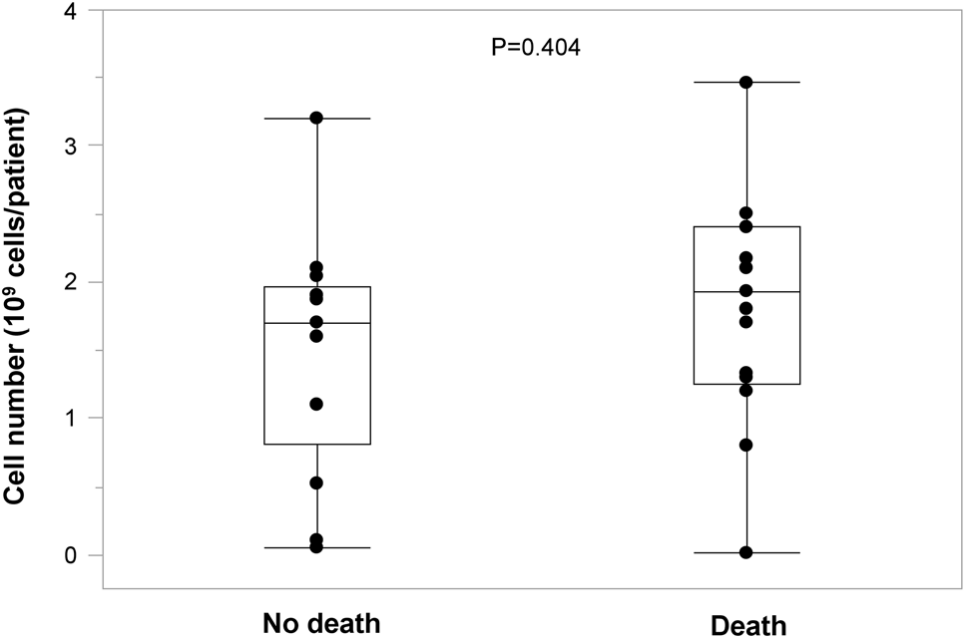
Box plots show implanted cell numbers in the no death and death groups.

Box plots show implanted cell numbers in the no death and death groups (Fig. 2). There was no significant difference in the numbers of cells implanted between the no death and death groups (1.5 ± 0.9 × 10^9^ vs. 1.8 ± 0.8 × 10^9^ cells per patient, P = 0.404).

### Implanted cell numbers in the MACE and no MACE groups

**Figure 3:**
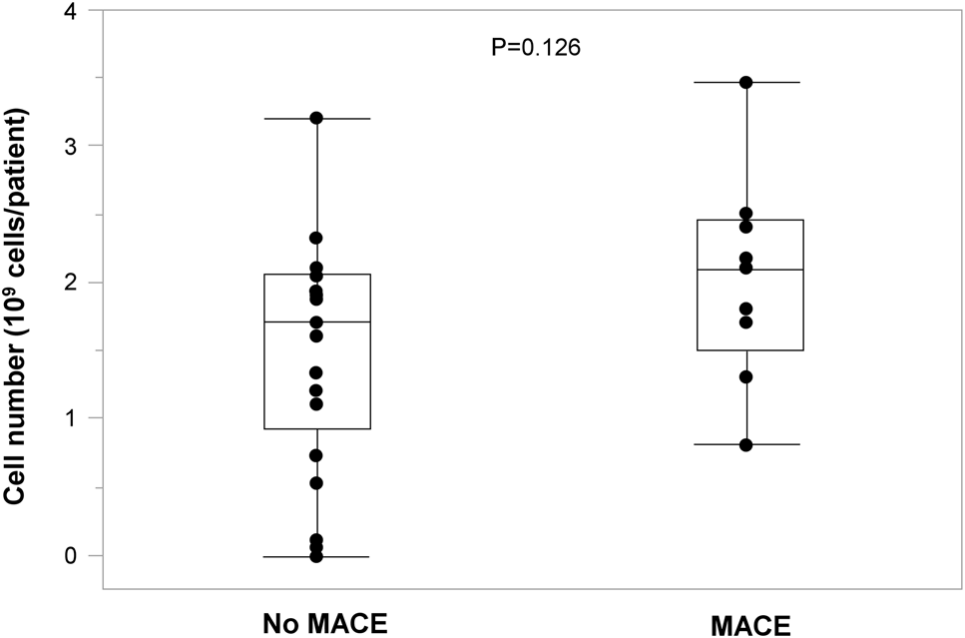
Box plots show implanted cell numbers in the MACE and no MACE groups.

Box plots show implanted cell numbers in the MACE and no MACE groups (Fig. 3). There was no significant difference in the numbers of cells implanted between the no MACE and MACE groups (1.5 ± 0.8 × 10^9^ vs. 2.0 ± 0.8 × 10^9^ cells per patient, P = 0.126).

### Discussion

The main goals for treatment of patients with PAD are to reduce the risk of cardiovascular outcomes, improve functional capacity, and preserve limb viability. In the present study, we demonstrated for the first time that the number of implanted BM-NMCs did not influence either the major amputation-free survival rate or overall survival rate in atherosclerotic PAD patients with CLI.

Protocols for therapeutic angiogenesis by cell therapy, gene therapy and other novel therapies (e.g., low-intensity pulsed ultrasound, granulocyte colony-stimulating factor and nanoparticle-mediated endothelial cell-selective drug delivery systems) have been developed with the aim of improving clinical symptoms in patients with CLI who have no option other than amputation^18^. Autologous BM-MNC implantation for CLI that results in increased collateral vessel formation and improvement of ischemic symptoms was first reported in 2002^8^. There have since been many studies on autologous cells derived from different sources and administered using different regimens for no-option CLI patients. Several lines of evidence have clearly shown that cell therapy including autologous BM-MNC implantation improves clinical symptoms and major amputation-free survival rate in atherosclerotic PAD patients with CLI. Recently, we have shown that the major amputation-free survival rate was higher in atherosclerotic PAD patients who underwent BM-MNC implantation than in internal controls and historical controls without cell therapy during a follow-up period of more than 10 years^11^. Next, we determined whether the difference in implanted cell number affects the major amputation-free survival rate in these patients. In the present study, there was no significant difference in implanted cell numbers between the no-amputation group and amputation group, suggesting that implanted cell numbers of 1.1 ± 0.7 × 10^9^ to 1.5 ± 0.8 × 10^9^ cells per limb did not affect the major amputation-free survival rate. In addition, these findings suggest that the number of implanted cells used in a clinical setting is sufficient to obtain good outcomes of limb salvage and subsequent survival. Additional analyses of cell number with a median number of 1.8 ± 0.8 × 10^9^ (range, 1.0 ± 0.8 × 10^7^—3.5 ± 0.8 × 10^9^) revealed that there was a significant difference between the ages of subjects in the small cell number and large cell number groups. The number of BM-MNCs obtained from bone marrow (BM) in younger subjects was significantly larger than that obtained from BM in older subjects. However, there were no significant differences between major amputation rates and mortality rates in the small cell number and large cell number groups. These findings suggest that younger subjects with larger implanted cell number may not contribute to the overall outcomes in this study population.

It is well known that the overall survival rate in atherosclerotic PAD patients with CLI is low and is almost the same as that in patients with advanced pancreatic cancer. It was expected that the overall survival rate in atherosclerotic PAD patients with CLI would be improved by cell therapy. However, previous studies, including our studies, have shown that cell therapy does not alter the mortality rate in atherosclerotic PAD patients with CLI^9,11,16,19^. In the present study, we confirmed that the implanted cell number also did not influence the mortality rate in atherosclerotic PAD patients with CLI who underwent BM-MNC implantation. There was also no significant difference in MACE between the two groups in the present study.

There is a number of limitations in this study. It was a retrospective observational trial performed in a single center. The number of subjects in the study was relatively small. The patients were referred to the University Hospital Vascular Function Study Group due to the severity of the disease, which rendered them unsuitable for conventional therapy. Dividing the subjects into separate groups of short-, mid- and long-term follow-up periods would further reduce the number of subjects per group for assessments. At present, we do not have sufficient results from data analysis to obtain definite conclusions. Due to the small number of subjects in this unique population, we were unable to further divide the subjects into groups. Further studies are needed to confirm the effects of BM-MNC implantation on overall outcomes in short-, mid- and long-term follow-up periods. In the present study, we evaluated the role of implanted cell number in clinical outcomes in atherosclerotic PAD patients with CLI. Assessment of the quality of implanted cells (e.g., cell proliferation, migration, apoptosis, and senescence and amount of angiogenic cytokines) would enable more specific conclusions concerning the role of BM-MNC implantation in the prevention of major amputation in atherosclerotic PAD patients with CLI to be drawn. The dose of BM aspirated from the ileum was based on the TACT trial protocol^8^. We could obtain 0.01 ± 0.7 × 10^9^ to 3.2 ± 0.7 × 10^9^ BM-MNCs for treatment per limb from 500 mL of BM. Under the condition of atherosclerotic PAD, we cannot obtain a large amount of BM. We cannot deny the possibility that implantation of a much larger number of BM-MNCs would have more beneficial effects on the prevention of major amputation and improve the mortality rate in atherosclerotic PAD patients with CLI. In addition, we cannot unfortunately obtain more detailed information on comorbidities other than death and major amputation during a long-term follow-up period.

## Conclusions

Differences in the number of BM-MNCs (mean number, 1.2 ± 0.7 × 10^9^ per limb; range, 0.01- 3.2 × 10^9^) derived from 500 mL of BM depending on the protocol of cell therapy did not alter the major amputation-free survival rate or mortality rate in atherosclerotic PAD patients with CLI. A larger number of BM-MNCs will not improve limb salvage outcome or mortality.

## Methods

### Study design

This study was a retrospective observational study. We previously reported amputation-free survival rates in atherosclerotic PAD patients with CLI who underwent BM-MNC implantation compared to those in internal controls and historical controls^11^. To identify limb survival projections as an internal control, the same limbs as those that were diagnosed with CLI and had no option for conventional treatments were estimated for amputation at the time when BM-MNC implantations were performed^11^. Additional data for the relationships of implanted cell number with updated overall survival, major amputation-free survival rates, and MACE in atherosclerotic PAD patients with CLI who underwent BM-MNC implantation were evaluated.

### Study subjects

Atherosclerotic PAD patients with CLI who had no-option for angioplasty or surgical revascularization were recruited for a BM-MNC implantation study performed between May 2002 and April 2014. Thirty Japanese patients with CLI were diagnosed after they complained of severe rest pain and non-healing ulcers. Statistical analyses were performed for 42 treated limbs in 30 atherosclerotic PAD patients with CLI. The diagnosis of arterial occlusion leading to ischemia was confirmed by angiography. Vasculitis and hypercoagulable states were ruled out. CLI was classified according to the guidelines of Tans-Atlantic Inter-Societal Consensus II^3^. Major amputation was defined as above the ankle amputation. The study protocol was previously approved by the Ethics Committee of Hiroshima University Graduate School of Biomedical Sciences, Hiroshima, Japan with Institutional Review Board number of E-10. The study was performed in accordance with the International Conference on Harmonization and Good Clinical Practice Guidelines implemented in Japan since 1997. Written informed consent for participation in the study was obtained from all patients during recruitment for participation in the clinical study and for follow-up progress data^20^.

### BM-MNC implantation

BM-MNCs were isolated and implanted in atherosclerotic PAD patients with CLI, as previously described^8^. In brief, 500 mL of bone marrow was aspirated from the ileum of a patient under general anesthesia and BM-MNCs were immediately isolated using a CS3000-Plus blood-cell separator (Baxter, Deerfield, IL) to obtain a final volume of 50 mL. One mL of BM-MNCs was implanted intramuscularly into each of a total of 40 sites with a 3 × 3-cm grid using a 22-gauge needle at a depth of 1.5 cm into the gastrocnemius of the ischemic leg.

### Statistical analysis

Results are presented as frequencies for categorical variables and means ± SD. All reported probability values were two-tailed. Values of *P* < 0.05 were considered significant. Continuous variables were compared between two groups by using the *t-*test. Categorical variables were compared by means of the chi-square test or Fisher’s exact test. The data analysis for this paper was generated using JMP® 13 for Macintosh. Copyright © 2016, SAS Institute Inc., Cary, NC, USA.
